# G1-like M and PB2 genes are preferentially incorporated into H7N9 progeny virions during genetic reassortment

**DOI:** 10.1186/s12917-021-02786-0

**Published:** 2021-02-15

**Authors:** Xiuli Li, Min Gu, Xiaoquan Wang, Ruyi Gao, Xinxin Bu, Xiaoli Hao, Jing Ma, Jiao Hu, Shunlin Hu, Xiaowen Liu, Sujuan Chen, Daxin Peng, Xinan Jiao, Xiufan Liu

**Affiliations:** 1 Animal Infectious Disease Laboratory, College of Veterinary Medicine, Yangzhou University, Yangzhou, Jiangsu China; 2 Jiangsu Co-innovation Center for Prevention and Control of Important Animal Infectious Diseases and Zoonosis, Yangzhou University, Yangzhou, Jiangsu China; 3 Jiangsu Key Laboratory of Zoonosis, Yangzhou University, Yangzhou, Jiangsu China; 4 Key Laboratory of Prevention and Control of Biological Hazard Factors (Animal Origin) for Agri-food Safety and Quality, Ministry of Agriculture of China, Yangzhou University, Yangzhou, Jiangsu China

**Keywords:** H7N9, H9N2, G1-like M, G1-like PB2, F/98-like M, F/98-like PB2, Advantage, Reassortment

## Abstract

**Background:**

Genotype S H9N2 viruses have become predominant in poultry in China since 2010. These viruses frequently donate their whole internal gene segments to other emerging influenza A subtypes such as the novel H7N9, H5N6, and H10N8 viruses. We recently reported that the PB2 and M genes of the genotype S H9N2 virus, which are derived from the G1-like virus, enhance the fitness of H5Nx and H7N9 avian influenza viruses in chickens and mice. However, whether the G1-like PB2 and M genes are preferentially incorporated into progeny virions during virus reassortment remains unclear; whether the G1-like PB2 and M genes from different subtypes are differentially incorporated into new virion progeny remains unknown.

**Results:**

We conducted a reassortment experiment with the use of a H7N9 virus as the backbone and found that G1-like M/PB2 genes were preferentially incorporated in progeny virions over F/98-like M/PB2 genes. Importantly, the preference varied among G1-like M/PB2 genes of different subtypes. When competing with F/98-like M/PB2 genes during reassortment, both the M and PB2 genes from the H7N9 virus GD15 showed an advantage, whereas only the PB2 gene from the H9N2 virus CZ73 and the M gene from the H9N2 virus AH320 displayed the advantage.

**Conclusion:**

Our findings highlight the preferential and variable advantages of H9N2-derived G1-like M and PB2 genes in incorporating them into H7N9 progeny virions over SH14-derived F/98-like M/PB2 genes.

**Supplementary Information:**

The online version contains supplementary material available at 10.1186/s12917-021-02786-0.

## Background

**Fig. 1:**
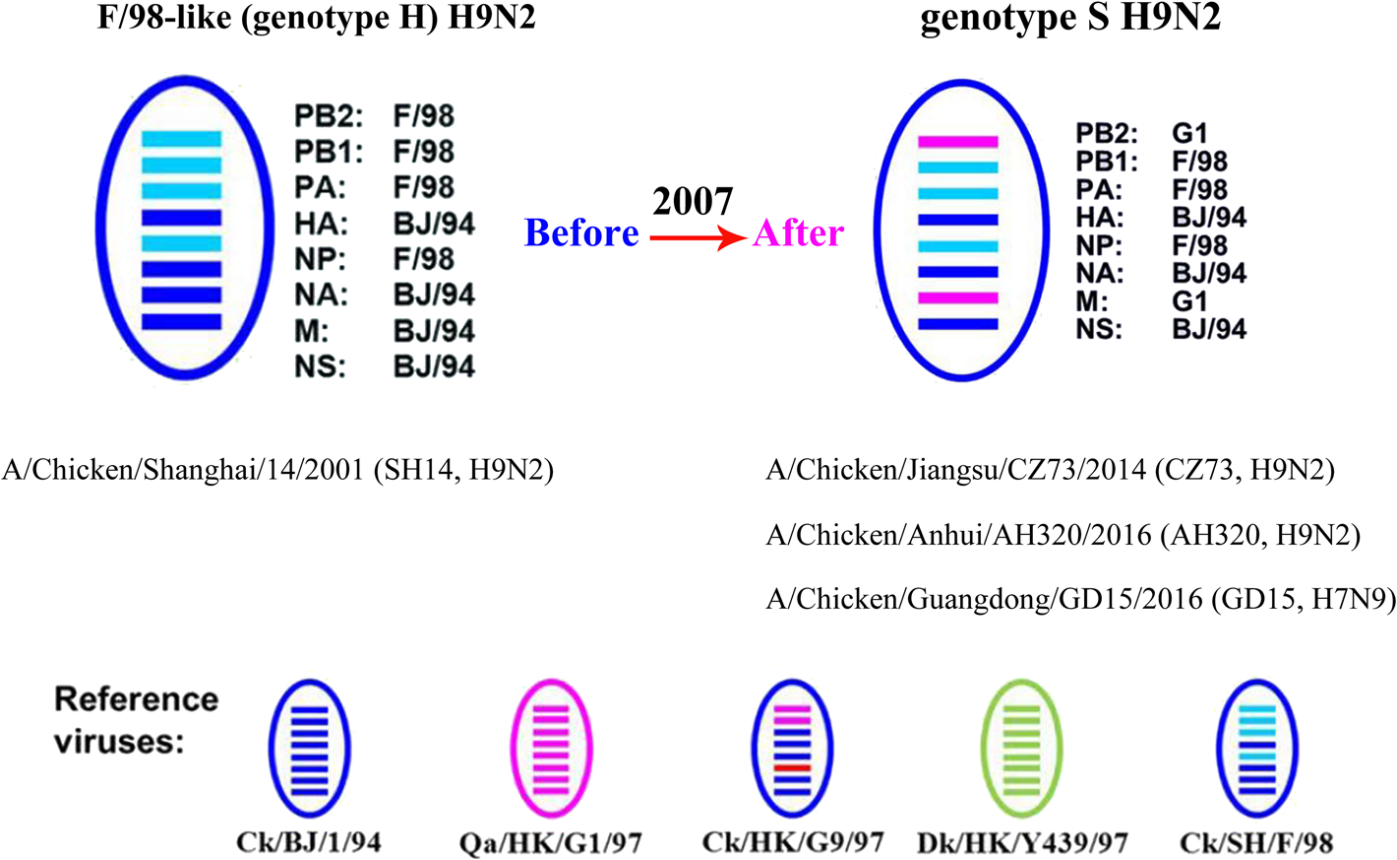
Schematic representation of the genetic constellations of the different viruses. SH14 virus were genotype H H9N2 virus; CZ73 and AH320 viruses were genotype S H9N2 viruses; and GD15 were H7N9 virus whose internal genes all came from genotype S H9N2 viruses

Widespread reassortment of H9N2 viruses in China has created various subtypes that can be phylogenetically grouped into the A-W genotypes [1]. More than one genotype may circulate simultaneously in one region. Some genotypes became dominant over a long period of time [2]. For instance, H9N2 viruses that harbour three polymerase genes and the NP gene from the F/98-like virus plus the remaining four genes from the BJ/94-like virus form a group of viruses designated as F/98-like or H genotype (Fig. 1) [2]. F/98-like (genotype H) viruses were first identified in 1998, became dominant in 2000, and persisted for several years thereafter [2, 3]. Since 2007, the genotype S viruses, which were generated through the reassortment of the F/98-like viruses (genotype H) by substituting their M and PB2 genes with those of the G1-like virus, have emerged (Fig. 1) and gradually dominated in chicken flocks after 2010 [2–5]. Moreover, genotype S H9N2 viruses often donate some or all the six internal genes to other emerging influenza A viruses in China [2, 6] such as the novel H7N9, H10N8, H7N7, and H5N6 viruses [2, 7–9]. That is, the H9N2 and H7N9 viruses currently circulating in China both possess G1-like M and PB2 genes [10].

The S genotype, which differs from F/98-like viruses (genotype H) only in their M and PB2 genes [11, 12], has not been replaced by a new genotype since 2010 [2]. As S genotype viruses carry the genetic backbone of F/98-like viruses (genotype H) plus the M and PB2 genes of the G1-like viruses, it is presumed that the G1-like M and PB2 genes confer better viral fitness over F/98-like counterparts.

Indeed, Pu et al. reported that H9N2 viruses with the G1-like M gene replicate faster in primary chicken embryonic fibroblasts and chickens than do the H genotype viruses. Furthermore, the H9N2 virus with the G1-like M gene exhibit an early surge of viral mRNA and genomic RNA production, suggesting of increased fitness of the virus [13]. Consistently, Hao et al. found that H5Nx and H7N9 viruses harbouring the G1-like PB2 and M genes display better viral fitness than those with F/98-like PB2 or M genes and have high virulence and replication capacity in chickens and mice [11, 12].

Our present study aims to determine if G1-like M and PB2 genes hold a competitive advantage during genetic reassortment, whether they play a role in maintaining the stability of “gene cassette” in H7N9 viruses. Several representative H7N9 and H9N2 viruses were chosen to test the relative copy number of G1-like M/PB2 genes and F/98-like M/PB2 genes in reassortant viruses on the H7N9 genetic background. The TaqMan-MGB quantitative realtime PCR (qRT-PCR) approaches for accurately quantifying the heteroplasmy level of G1-like M/PB2 and F/98-like M/PB2 were introduced in our study. The MGB probes had higer melting temperature. Therefore, they are much shorter than the traditional TaqMan probes, which makes MGB probes more sensitive to single base mismatches [14, 15]. Multiple studies have demonstrated that TaqMan-MGB qRT-PCR is an accurate technique with high specificity, sensitivity and remarkable reproducibility and is quite attractive for use in SNP (single nucleotide polymorphism) detection and allelic discrimination [16, 17].

Our results suggests that the G1-like M and PB2 genes are more likely to be incorporated into the novel H7N9 viruses than that of SH14 virus derived F/98-like M/PB2 genes; G1-like PB2/M genes derived from different virus strains display variable competitive advantages during virus reassortment.

## Results

### The sensitivity and specificity of duplex real-time RT-qPCR assay

We first evaluated the sensitivity of duplex RT-qPCR by using ten-fold serially diluted plasmid mixtures as the templates in the amplification reaction. As shown in Supplementary Fig. S1A & B and Fig. S2A & B, each gene could be readily amplified with approximately 10 copies of templates when crossing point was less than 35(cp < 35). The standard curves revealed excellent correlation coefficient and amplification efficacy (Fig. S1 C-F, Fig. S 2 C-F and Fig. S 3 A-D) when cp < 35. Moreover, there was no significant difference in amplification efficiency among the probes. To determine the specificity of duplex RT-qPCR used in this study, the G1-like M/PB2 plasmids from GD15, CZ73, AH320 viruses and the F/98-like M/PB2 plasmids from genotype H H9N2 SH14 virus were used as templates for the amplification reactions. As shown in Supplementary Fig. S4, the M/PB2 genes from GD15, CZ73 and AH320 could not be detected with SH14-M_probe974_ or SH14-PB2_probe713_; whereas M/PB2 genes of SH14 virus could not be detected by using GD15-M_probe974_, M_probe975–17_, GD15-PB2_probe713_ or PB2_probe974RC_.

### G1-like M and PB2 genes from GD15 (H7N9) virus hold a competitive advantage during reassortment

**Fig. 2:**
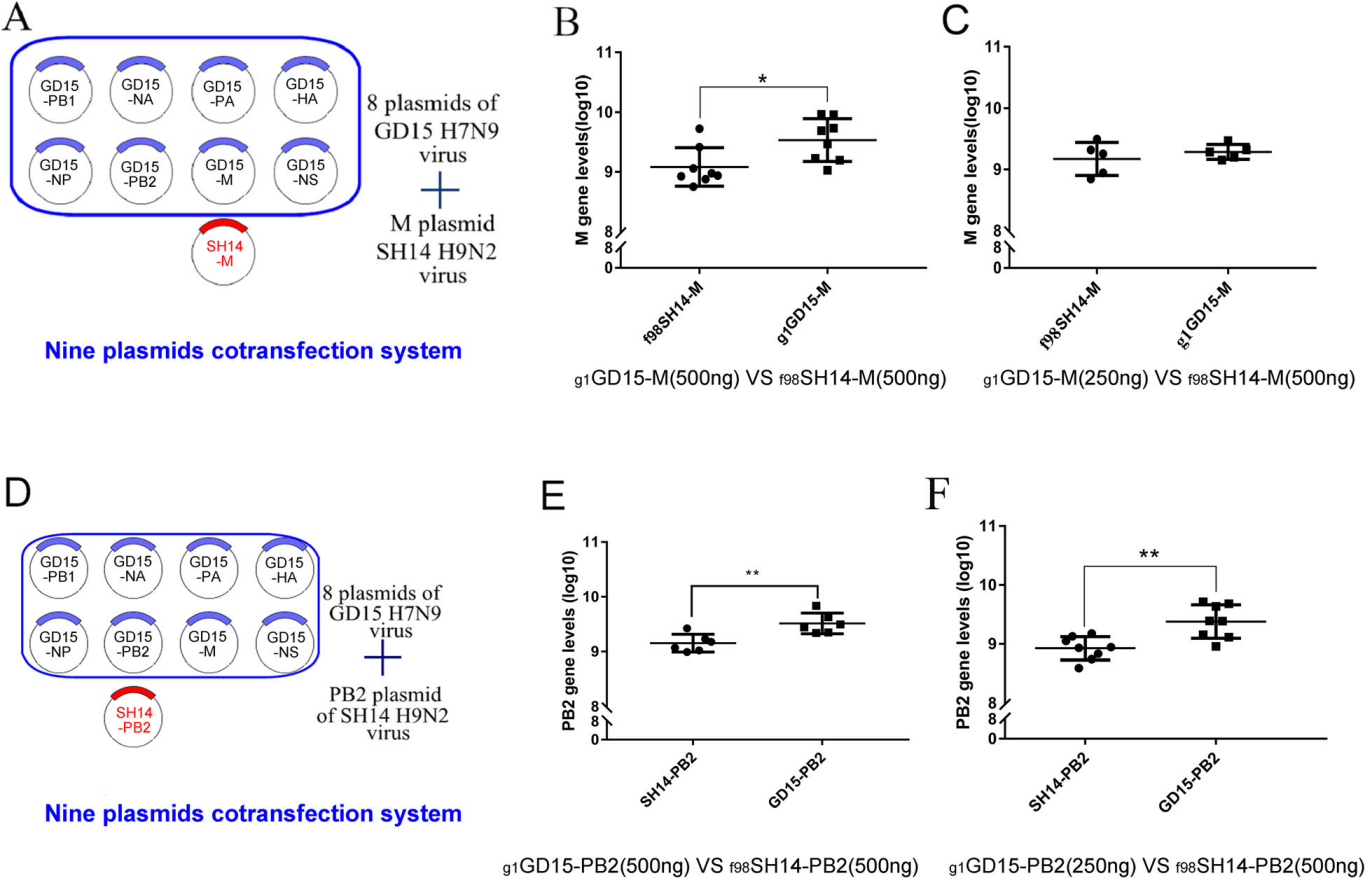
Competitive between _**g1**_GD15-M/PB2 and _**f98**_SH14-M/PB2 genes during cotransfection. (A & D) Schematic representation of the plasmids, and different amount of M and PB2 plasmids were used in the cotransfections. (B&C) Quantitative analysis of _**g1**_GD15-M and _**f98**_SH14-M genes in virus population expanded from the contransfection. 293 T cells were cotransfected with nine plasmids, supernatants were collected and inoculated into eggs. qRT-PCR was performed to determine the numbers of _**g1**_GD15-M and _**f98**_SH14-M genes. (E & F) Quantitative analysis of _**g1**_GD15-PB2 and _**f98**_SH14-PB2 genes in virus population expanded from the contransfection. 293 T cells were cotransfected with nine plasmids, 500 ng (E) and 250 ng (F) of _**g1**_GD15-PB2 plasmid was used respectively. Supernatants were collected and inoculated into eggs. qRT-PCR was performed to determine the numbers of _**g1**_GD15-M and _**f98**_SH14-M genes in allantoic fluid. Data are represented as mean ± SD (*N* = 8). The statistically significant differences were analyzed by ANOVA (*P < 0.05; **P < 0.01; ***P < 0.001)

To determine whether the G1-like M segment was preferentially incorporated into reassorted virus progeny when it was in competition with F/98-like M gene, 293 T cells were co-transfected with eight plasmids of the GD15 virus plus the ninth one encoding _**f98**_SH14-M gene (500 ng /plasmid) (Fig. 2a) [18]. After incubation for 72 h, the conditioned media of transfected cells, which contained the reassorted progeny virions, were used to inoculate into embryonated chicken eggs. Quantitative RT-PCR (qRT-PCR) analysis of allantoic fluids revealed that the copy number of the _**g1**_GD15-M gene was significantly greater than that of the _**f98**_SH14-M gene (Fig. 2b). However, when just 250 ng of the _**g1**_GD15-M plasmid was repeated for the above mentioned 9-plasmid transfection, approximate level of gene copies was displayed between _**g1**_GD15-M and _**f98**_SH14-M (Fig. 2c).

We next determined whether the G1-like PB2 gene also exhibited competitive advantage during genetic reassortment. Co-transfection experiments with the nine-plasmid system were similarly carried out as above. As shown in Fig. 2d, the copy number of the _**g1**_GD15-PB2 gene was significantly higher than that of the _**f98**_SH14-PB2 gene in the viruses rescued from the cells transfected with 500 (Fig. 2e) or 250 ng (Fig. 2f) plasmid each. The competitive advantage of the G1-like PB2 gene from GD15 virus was more prominent than that of the G1-like M gene.

### Variable advantage of G1-like M/PB2 genes from different strains during reassortment

**Fig. 3:**
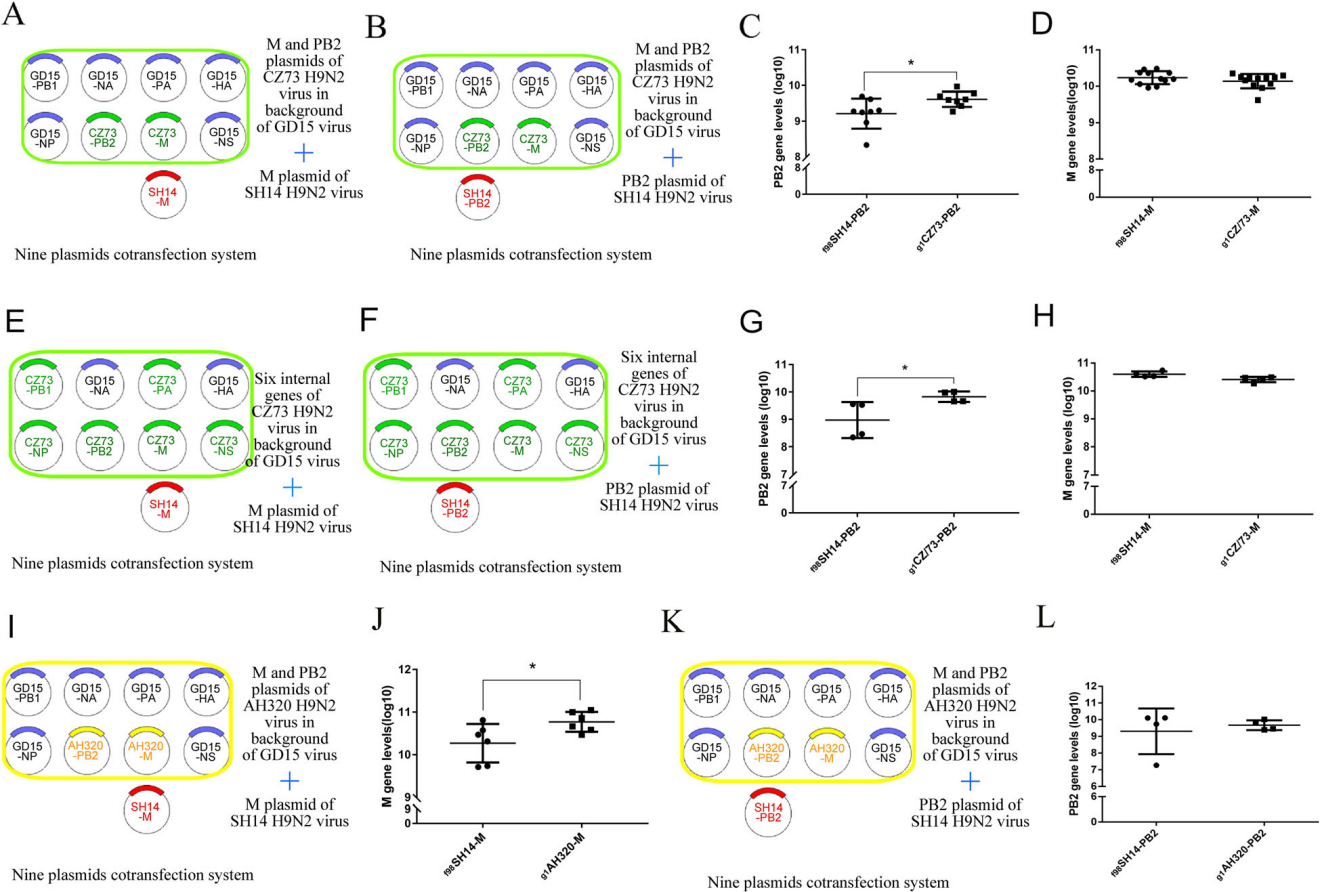
Schematic representation of the system used to perform cotransfection and coinfection. (A & B) Competitive between _**g1**_CZ73-M/PB2 and _**f98**_SH14-M/PB2 in backgroud of GD15 with PB2 and M from CZ73 and M/PB2 from SH14 during cotransfection. (E & F) Competitive between _**g1**_CZ73-M/PB2 and _**f98**_SH14-M/PB2 during cotransfection, where the internal genes of GD15 were replaced by those of CZ73. (I & K) Competitive between _**g1**_AH320-M/PB2 and _**f98**_SH14-M/PB2 in backgroud of GD15 with PB2 and M from AH320 and M/PB2 from SH14 during cotransfection. 500 ng of each plasmid was used in above cotransfections. (C & D & G & H & J & L) Quantitative analysis of PB2 and M genes in allantoic fluid. Data are represented as mean ± SD (*N* = 6). The statistically significant differences were analyzed by ANOVA (*P < 0.05; **P < 0.01; ***P < 0.001)

We next investigated if the competitive advantage of the M and PB2 genes from G1-like viruses in reassortment was strain-specific. The G1-like M and PB2 genes from two additional S genotype H9N2 viruses, A/Chicken/Jiangsu/CZ73/2014 (CZ73) and A/Chicken/Anhui/AH320/2016 (AH320), were used in co-transfection experiments as described above. As shown in Fig. 3a, b, the copy number of the _**g1**_CZ73-PB2 gene was significantly higher than that of the _**f98**_SH14-PB2 gene in the rescued viruses (Fig. 3c). However, there was no significant difference in the copy number of the M gene in reassortant viruses rescued from co-transfection with the _**g1**_CZ73-M and _**f98**_SH14-M genes (Fig. 3d). We then investigated the effect of the internal gene cassette of the CZ73 virus on virus reassortment. Co-transfections with six plasmids encoding the internal genes of CZ73 virus plus the plasmid encoding the _**f98**_SH14-PB2 or _**f98**_SH14-M (500 ng/plasmid) (Fig. 3e, f) revealed that the copy number of _**g1**_CZ73-PB2 genes was significantly higher than that of _**f98**_SH14-PB2 gene (Fig. 3g), although _**g1**_CZ73-M genes did not exhibit any competitive advantages (Fig. 3h).

We then conducted a similar co-transfection experiment by using the M or PB2 gene of another S genotype H9N2 virus, AH320 (Fig. 3i, k). qPCR analysis revealed that the copy number of the _**g1**_AH320-M gene in the rescued viruses was significantly higher than that of the _**f98**_SH14-M gene (Fig. 3j). The copy number of the _**g1**_AH320-PB2 gene in rescued viruses was higher than that of the _**f98**_SH14-PB2 gene. However, this was not statistically significant (Fig. 3l).

### Lack of competitive advantage for the _g1_GD15-M gene incorporation into the H7N9 background over _f98_SH14-M gene during co-infection

**Fig. 4:**
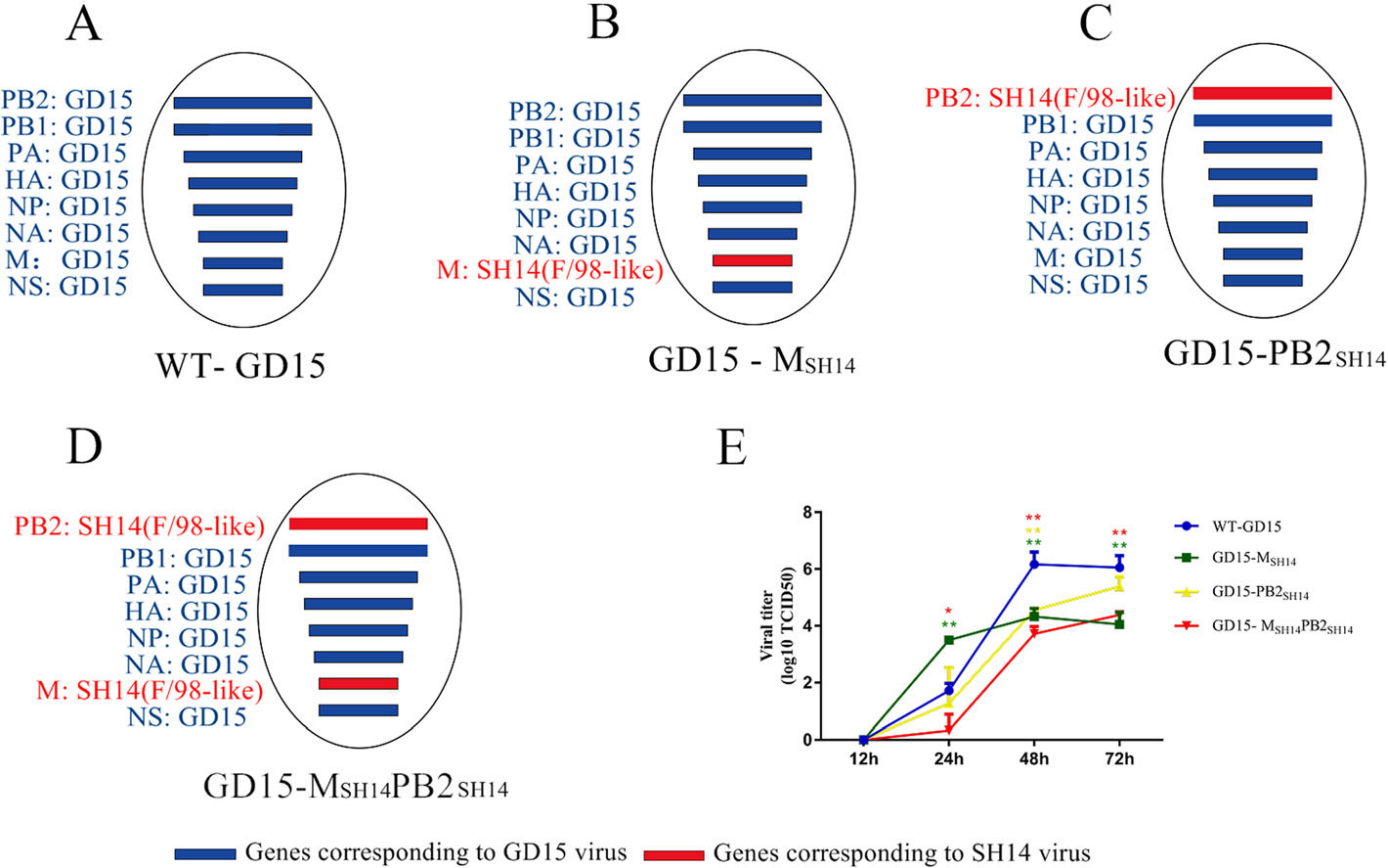
Schematic representation of reassortant viruses and their growth kinetics. (A) Reassortant virus generated from eight plasmid of GD15 virus. (B) Reassortant virus bearing the genetic backbone of GD15 virus, with the _**g1**_GD15-M gene being replaced by _**f98**_SH14-M gene. (C) Reassortant virus bearing the genetic backbone of GD15 virus, with the _**g1**_GD15-PB2 being replaced by _**f98**_SH14-PB2 gene. (D) Reassortant virus bearing the genetic backbone of GD15 virus, with _**g1**_GD15-M and _**g1**_GD15-PB2 being replaced by with _**f98**_SH14-M and _**f98**_SH14-PB2 genes. (E) The growth Kinetics of the reassortant viruses in MDCK cells. MDCK cells were infected with virus at an MOI of 0.01. At the indicated times after infection, virus titeres in the supernatant were determined. Data represent the mean ± SD (*n* = 3) from triplicate independent infections. The statistically significant differences were analyzed by ANOVA compared with WT-GD15 virus (*P < 0.05; **P < 0.01; ***P < 0.001)

**Fig. 5:**
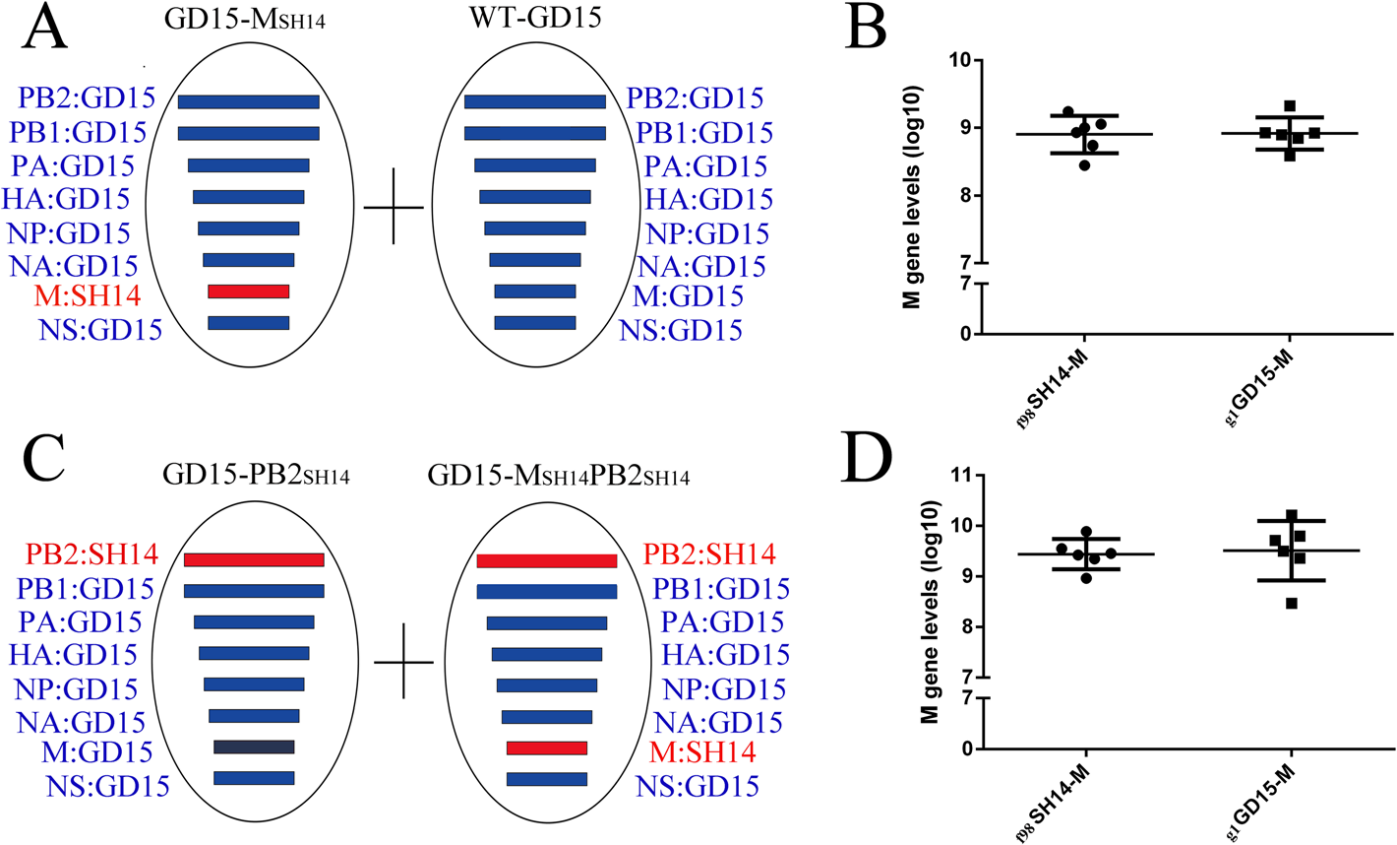
Competitive between _**g1**_GD15-M and _**f98**_SH14-M genes during coinfection. (A) MDCK cells were coinfected with GD15-M_SH14_ and WT-GD15 viruses at the MOI of 0.01 (C) MDCK cells were infected with GD15-PB2_SH14_ and GD15-M_SH14_PB2_SH14_ viruses at the same MOI of 0.01; (B & D) Quantitative analysis of _**g1**_GD15-M and _**f98**_SH14-M genes in supernatants of the coinfected cells by qRT-PCR. Data are presented as mean ± SD (n = 3). The statistically significant differences were analyzed by ANOVA (*P < 0.05; **P < 0.01; ***P < 0.001)

We next determined if the competitive advantage of G1-like M and PB2 genes during reassortment was in part due to faster replication of newly reassorted viruses. We generated four recombinant viruses (WT-GD15, GD15-M_SH14_, GD15-PB2_SH14_ and GD15-M_SH14_PB2_SH14_) (Fig. 4a-d). As shown in Fig. 4e, the titers of WT-GD15 virus at 48 h post infection (h.p.i) were significantly higher than three recombinant viruses, which gave similar virus titers in the conditioned media. Quantitative RT- PCR analysing the M gene revealed almost equal _**g1**_GD15-M and _**f98**_SH14-M vRNA levels at 48 hpi (Table 1). We then co-infected MDCK cells with GD15-M_SH14_ and WT-GD15 viruses, each with 0.01 MOI (Fig. 5a). Again, quantitative RT- PCR revealed that the levels of the _**g1**_GD15-M and _**f98**_SH14-M genes in the conditioned media were not significantly different (Fig. 5b). To further investigate whether PB2 genes affected the competitive advantage of M genes at the virus level, we analysed the portion of _**g1**_GD15-M gene in progeny virions by co-infecting MDCK cells with recombinant GD15-PB2_SH14_ and GD15-M_SH14_PB2_SH14_ viruses (Fig. 5c). However, the copy number of _**g1**_GD15-M genes in progeny virions did not demonstrate significantly advantage over the _**f98**_SH14-M gene (Fig. 5d).

### Preference for the _g1_GD15-PB2 gene incorporation into the H7N9 background over _f98_SH14-PB2 gene during co-infection

**Fig. 6:**
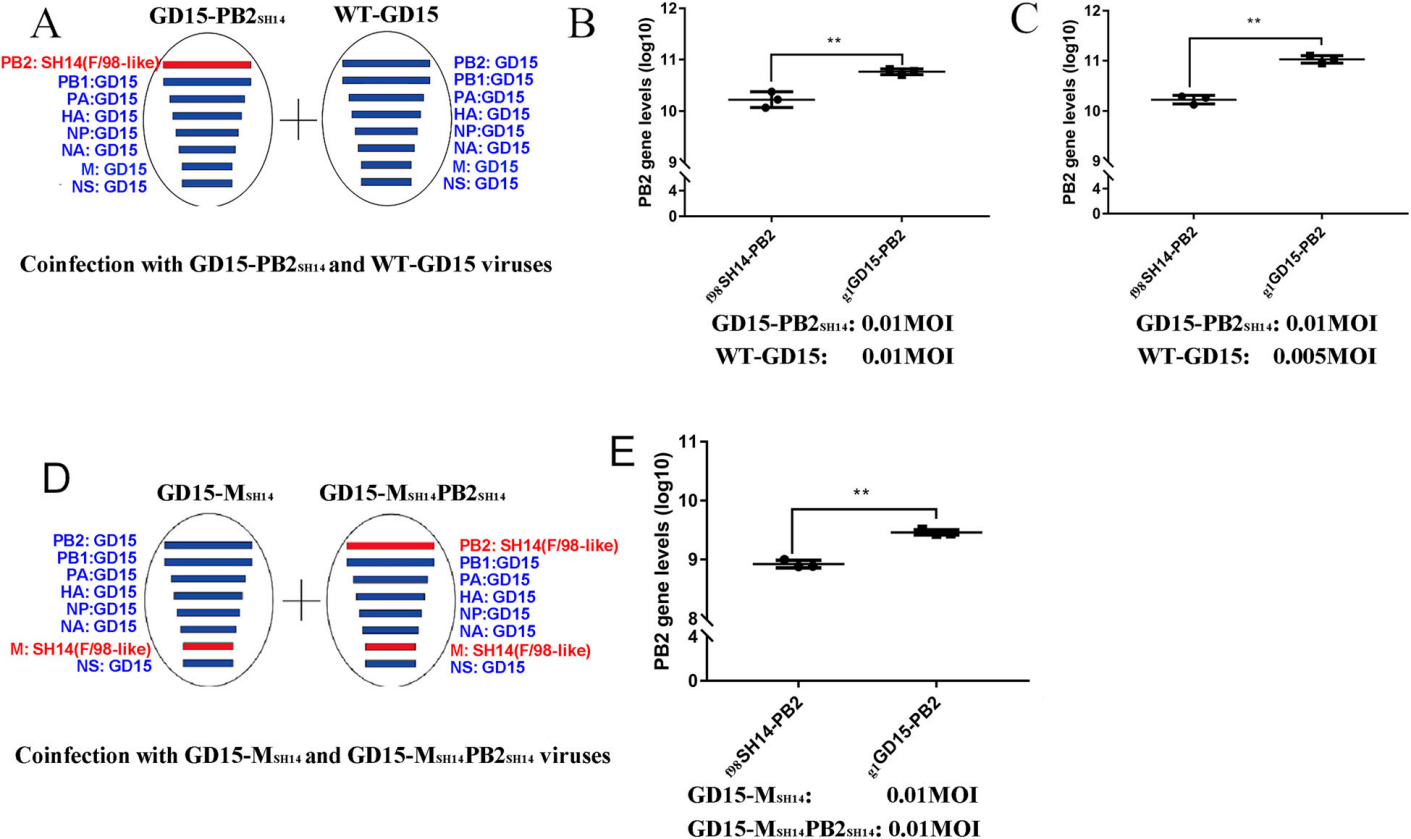
Competitive between _**g1**_GD15-PB2 and _**f98**_SH14-PB2 genes during coinfection. (A) Schematic representation of coinfection experiment. MDCK cells were infected with GD15-PB2_SH14_ at the MOI of 0.01 and WT-GD15 viruses at the MOI of 0.01 or 0.005; (D) Schematic representation of coinfection experiment. MDCK cells were infected with GD15-M_SH14_ and GD15-M _SH14_PB2_SH14_ viruses at the same MOI of 0.01; (B, C & E) Quantitative analysis of _**g1**_GD15-PB2 and _**f98**_SH14-PB2 genes in supernatants of the coinfected cells by qRT-PCR. Data are presented as mean ± SD (n = 3). The statistically significant differences were analyzed by ANOVA (*P < 0.05; **P < 0.01; ***P < 0.001)

MDCK cells were co-infected with GD15-PB2_SH14_ and WT-GD15 viruses (0.01 MOI each) (Fig. 6a). qRT-PCR analysis revealed that the levels of the G1-like PB2 gene in the progeny viruses was significantly higher than that of the F/98-like PB2 gene (Fig. 6b). Given that these two viruses replicate differentially, 0.005 moi WT-GD15 virus was used in co-infection experiments. We found that at this dosage the two PB2 genes replicated at a comparable rate at 48 hpi (Table 1). Besides, another conserved gene-NP segment were detected to verify whether the changes in the levels of M and PB2 are true. The results showed that at this dosage the number of NP gene copies in progeny viruses were similar among the reassortant viruses, too (Table 1). In the coinfection experiment the number of WT-GD15 virus used was only half of that GD15-PB2_SH14_, the _**g1**_GD15-PB2 gene still demonstrated significant advantages (Fig. 6 C).

Since genotype S H9N2 strains are generated through the replacement of the M and PB2 genes of F/98-like viruses with those from the G1-like viruses, we wondered if the M or PB2 genes would influence each other’s preference. MDCK cells were coinfected with GD15-M_SH14_ and GD15-M_SH14_PB2_SH14_ viruses (Fig. 6 D). The copy number of the _**g1**_GD15-PB2 gene were significantly higher than that of _**f98**_SH14-PB2 genes in the progeny viruses (Fig. 6e). However, the _**g1**_GD15-PB2 to _**f98**_SH14-PB2 ratio in this co-infection experiment was similar to that co-infected with _**g1**_GD15-PB2_SH14_ and WT-GD15 viruses (date not shown), suggesting that the competitive advantage of _**g1**_GD15-PB2 to _**f98**_SH14-PB2 during reassortment is not influenced by the M gene.

## Discussion

If two homologous gene segments are available in a cell, they will compete with other for incorporation into progeny viruses [19]. We performed co-transfection and co-infection experiments with the M or PB2 gene derived from the G1-like and F/98-like genotypes. During reassortment, the M or PB2 gene from two genotypes would compete with other for their incorporation into progeny virions. Our co-transfection experiments showed that the copy number of _**g1**_GD15-M/ _**g1**_GD15-PB2 genes were higher in progeny virions than that of _**f98**_SH14-M/PB2 gene. These observations suggest that there is a biased genetic reassortment between G1-like M/PB2 and F/98-like M/PB2 genes. The advantage of the _**g1**_GD15-PB2 but not _**g1**_GD15-M gene in incorporating into progeny virions was conformed in our co-infection experiment. The discrepancy in the results obtained from co-transfection and co-infection experiments are likely due to the differences in the materials and methods used in the study. In addition, the interactions between viruses are more complex than plasmids. Consistent with our observations, Essere and Kawaoka reported that co-transfection and co-infection result in different reassortant genotypes [18, 20, 21]. These authors postulated that some unknown cellular factors may affect genetic reassortment [18].

The advantage of the G1-like M/PB2 genes over that of the F98-like M/PB2 genes were further investigated by using two additional genotype S H9N2 viruses, CZ73 and AH320. We found that the incorporation advantage of the G1-like M/PB2 gene was variable among strains. The findings that only the PB2 gene from the H9N2 virus CZ73 and the M gene from the H9N2 virus AH320 had an advantage suggest that the competitive advantage is not equal for the M and PB2 genes from one virus. We speculate that the internal genes of a novel H7N9 virus are not necessarily from one H9N2 virus but rather from a super recombination of genes from different H9N2 viruses.

The segmented genome of influenza viruses allows for the reassortment of gene segments between viruses when they co-infect same cells [22, 23], resulting in multiple genotypes of influenza viruses [23–25]. Nevertheless, the substitution of the G1-like M and PB2 genes has reduced the genetic diversity of H9N2 viruses [8, 13]; S genotype H9N2 viruses have been predominant in chickens since 2010 [2–4]. We speculate that the uniqueness of the internal gene cassette of the S genotype makes it possible to reach a more ideal equilibrium.

Our study has demonstrated an advantage of G1-like M and PB2 genes over F/98-like M and PB2 genes. It is not clear why the internal genes of the S genotype H9N2 virus remain stable as a cassette and has stayed prevalent in H9N2 and H7N9 viruses for many years. The genotype S H9N2 viruses provide their internal genes to various emerging viruses, especially H7N9 viruses [1, 5, 10, 13, 26]. Epidemiological evidence suggests that nearly all human and avian H7N9 isolates possess internal genes that originated from H9N2 viruses [8, 27, 28]. It is likely that the six internal genes of genotype S H9N2 viruses have reached a stable and optimal combination, ensuring the donation of the internal gene cassette to emerging viruses. On the other hand, we also observed gene segments that originated from H9N2 viruses in other viruses, such as H5N2 and H7N7 viruses but not as a whole cassette [4]. The uniqueness of internal genes of H9N2 viruses, especially those of genotype S, warrants further exploration.

## Conclusions

Our study has demonstrated that G1-like PB2/M genes had competitive advantages over SH14 virus-derived F/98-like PB2/M gene during reassortment. However, their competitive advantage varied among different strains. The competitive advantage of the PB2 gene was more prominent than that of the M gene. Our results suggest that the preferential incorporation of H9N2-derived G1-like M and PB2 genes into progeny virions of H7N9 influenza viruses may help maintain the stability of the internal gene cassette in the novel H7N9 viruses.

## Methods

### Cells, eggs and viruses

Human embryonic kidney (293 T) cells and Madin-Darby canine kidney (MDCK) cells were stored by our laboratory and maintained in DMEM supplemented with 10% foetal bovine serum (FBS) and penicillin-streptomycin. The SPF embryonated chicken eggs were purchased from Beijing Merial Vital Laboratory Animal Technology Company.

The H7N9 viruses A/Chicken/Guangdong/GD15/2016 (GD15, whose internal genes are all genotype S, Fig. 1), the S genotype H9N2 viruses A/Chicken/Jiangsu/CZ73/2014 (CZ73, Fig. 1) and A/Chicken/Anhui/AH320/2016 (AH320, Fig. 1) and the H genotype H9N2 viruses A/Chicken/Shanghai/14/2001 (SH14, Fig. 1) used in this study were isolated and preserved by our laboratory.

### Plasmid construct

The 8-plasmid reverse genetic systems of GD15(H7N9) virus, CZ/73(H9N2) virus and AH320(H9N2) virus were constructed in the present study, while the 8 plasmids for SH14(H9N2) viruswas constructed as previously report [29]. All constructs were verified by sequencing and preserved by our laboratory.

### Nine plasmid co-transfection

Six plasmids containing PB1, PA, NP, NS, HA, and NA genes of GD15 (H7N9) virus, plus PB2/M plasmid from GD15/CZ73/AH320 virus and PB2/M plasmid from SH14 (F98-like H9N2) virus were cotransfected into 293 T and MDCK cells to examine the competition between G1-like and F/98-like PB2. The supernatants were collected after 72 h for inoculation in 9–10 day-old egg. One egg was used for each sample. 36 h post inoculation the allantoic fluid was collected and stored in − 70 °C.cotransfection experiment was run in triplicate wells and repeated at least twice for each sample.

### Reverse genetics

Reassortant viruses were generated by reverse genetics as previously described [30]. Plasmids were transfected into 293 T and MDCK cells using Polyfect transfection reagent (Qiagen) according to the manufacturer’s instructions. After 72 h, supernatants were harvested and each of the newly generated viruses was plaque purified. The purified viruses were amplified on SPF embryonated chicken eggs to generate the viral stock used in the study.

### Viral growth kinetics

The growth properties of reassortant viruses were assessed as follows. Triplicate wells containing MDCK cells were infected with the indicated viruses at a multiplicity of infection (MOI) of 0.01, supplemented with Opti-MEM (catalog no. 31985–070; Gibco) and incubated at 37 °C. Supernatants were collected from each well at 12, 24, 48 and 72 h post infection (h.p.i.) and were stored at − 70 °C. Viral titres were subsequently determined as the 50% tissue culture infection dose (TCID50) per 0.1 ml in MDCK cells using the method of Reed and Muench [31].

### Coinfection of MDCK cells with Reassortant viruses

MDCK cells were coinfected with viruses at 0.01 or 0.005 MOIs. After incubation at 37 °C for 1 h, the virus inoculum was removed, and the cells were washed three times with phosphate-buffered saline (PBS), following by incubation at 37 °C in Opti-MEM. After 48 h, supernatants were collected from each well for subsequent analysis. Every experiment was run in triplicate wells and repeated at least twice.

### Quantitative real-time RT-PCR screening of gene origins

Total RNA was extracted from the allantoic fluid or cell culture supernatant using a Solarbio RNA extraction kit (Solarbio, Shanghai, China). And the extracted RNA for all samples were diluted to 100 ng/ul to guarantee equal amount of total RNA was used for the downstream RT-PCR. DNA was cleared by digesting 2 μL of vRNA with 4 μL of 4× gDNA wiper Mix (Vazyme Biotech, Nanjing, China) and 10 μL of water for 2 min at 42 °C. Next, unique 12 bp primer (5′-AGCAAAAGCAGG-3′) was used to perform reverse transcription. To identify gene origins, we designed a pair of primers (M-F948, and M-R1012) in the same sequences of _**f98**_SH14-M, _**g1**_GD15-M, _**g1**_CZ73-M and _**g1**_AH320-M, as well as three MGB probes, including SH14-M_probe974_ (against _**f98**_SH14-M), GD15-M_probe974_ (against _**g1**_GD15-M), and M_probe975–17_ (against _**g1**_CZ73-M and _**g1**_AH320-M) in the different sequences of _**f98**_SH14-M, _**g1**_GD15-M, _**g1**_CZ73-M and _**g1**_AH320-M between the two primers. Three probes were used for quantitative real-time RT-PCR to quantitatively detect gene copies of _**f98**_SH14-M, _**g1**_GD15-M, _**g1**_CZ73-M, and _**g1**_AH320-M. Likewise, we designed a pair of primers and three MGB probes, PB2-F650, PB2-R772, SH14-PB2_probe713_, GD15-PB2_probe713_ and PB2_probe974RC_ (against _**g1**_CZ73-PB2 and _**g1**_AH320-PB2), to quantify copies of _**f98**_SH14-PB2, _**g1**_GD15-PB2, _**g1**_CZ73-PB2 and _**g1**_AH320-PB2. The specificity of each probe has been verified as early as we received the products. The sequences of the primers and probes are shown in Table 2.

**Table 1:** Number of copies of M/PB2 gene (log_10_)

Reassortant virus	WT-GD15 (0.01 MOI)	WT-GD15 (0.005 MOI)	GD15-PB2_SH14_ (0.01 MOI)	GD15-M_SH14_ (0.01 MOI)	GD15-M_SH14_PB2_SH14_ (0.01 MOI)
Copies of PB2 gene (log_10_)	11.14 ± 0.15	10.33 ± 0.06	10.29 ± 0.06		10.56 ± 0.05
Copies of M gene (log_10_)	11.18 ± 0.12	10.74 ± 0.20		11.37 ± 0.10	11.11 ± 0.07
Copies of NP gene (log_10_)		9.92 ± 0.35	9.83 ± 0.09	10.16 ± 0.10	9.68 ± 0.36

MDCK cells were infected with each virus alone at 0.01 or 0.005 MOI and incubated for 48 h. The replication level of PB2, M and NP gene were detected by qRT-PCR. Results represent the average copies from three independent infections

**Table 2:** Primers and MGB probes used in qPCR in this study and targeting genes they used to detect

Primer /probe	Sequence(5′ to 3′)	Gene detected
M-F_948_	TGCCTGAGTCTATGAGGGAAG	GD15-M, SH14-M, CZ73-M, AH320-M
M-R_1012_	ACCATCGTCAACATCCACAG	GD15-M, SH14-M, CZ73-M, AH320-M
SH14-M_probe974_	FAM-CGGCAGGAGCAACAGAG-MGB	SH14-M
GD15-M_probe974_	VIC-CAGCATTCTGCCGTTCCT-MGB	GD15-M
M_probe975–17_	VIC-AGCATTCTGCTGTTCCT-MGB	CZ73-M, AH320-M
PB2-F_650_	TTGCTCCTTTAATGGTGGC	GD15-PB2, SH14-PB2, CZ73-PB2, AH320-PB2
PB2-R_772_	AGGTCCCTTGAGTCAAATGC	GD15-PB2, SH14-PB2, CZ73-PB2, AH320-PB2
SH14-PB2_probe713_	FAM-CGGTAGCAGGTGGAACAA-MGB	SH14-PB2
GD15-PB2_probe713_	VIC-CAGTGGCTGGAGGGACA-MGB	GD15-PB2
PB2_probe974RC_	VIC-TTGTCCCTCCAGCTACTGG-MGB	CZ73-PB2, AH320-PB2

Quantitative real-time PCR was carried out in a 20 μL reaction mixtures containing 1 μL of cDNA, 250 nM each primer, 100 nM FAM probes, 100 nM VIC probe, 10 μL of 2× AceQ U+ Probe Master Mix (Vazyme Biotech, Nanjing, China), and water. The qPCR conditions were as follows: 37 °C for 2 min and 95 °C for 5 min, followed by 45 cycles of 95 °C for 10 s, 60 °C for 30 s and 40 °C for 2 min. The plasmids pHW2000-M_SH14_, pHW2000-M_GD15_, pHW2000-M_CZ73_, pHW2000-M_AH320_, pHW2000-PB2_SH14_, pHW2000-PB2_GD15_, pHW2000-PB2_CZ73_ and pHW2000-M_AH320_, were used as standards for the M/PB2 genes of SH14, GD15, CZ73 and AH320 viruses, respectively.

### Statistical analysis

Statistical analyses were conducted by using SAS software, version 9.2 (SAS Institute).Statistically significant differences between the number of the copies of G1-like M/PB2 and F/98-like M/PB2 genes were analysed by using Duncan’s multiple range test in ANOVA. Differences were considered significant at *P* < 0.05.

## Supplementary Information

**Additional file 1 **: **Figure S1.** Sensitivities and standard curves of the duplex TaqMan-MGB qRT-PCR targeting the M genes of SH14 and GD15 viruses. Sensitivities of SH14-M_probe974_ (A) and GD15-M_probe974_ (D) were detected by 10-fold serial dilutions of 10^8^copies/ml _f98_SH14-M plasmid and _g1_GD15-M plasmid, respectively. The detection limit was approximately 10 copies of both gene when cp < 35. The amplification curves and the corresponding standard curve for detection of _f98_SH14-M (B, C) and _g1_GD15-M (E, F) showed excellent efficiencies of the duplex TaqMan-MGB qRT-PCR.

**Additional file 2 **: **Figure S2.** Sensitivities and standard curves of the duplex TaqMan-MGB qRT-PCR targeting the PB2 genes of SH14 and GD15 viruses. Sensitivities of SH14-PB2_probe713_ (A) and GD15-PB2_probe713_ (D) were detected by 10-fold serial dilutions of 8*10^8^copies/ml _f98_SH14-PB2 plasmid and _g1_GD15-PB2 plasmid, respectively. The detection limit was approximately 10 copies of both gene when cp < 35. The amplification curves and the corresponding standard curve for detection of _f98_SH14-PB2 (B, C) and _g1_GD15-PB2 (E, F) showed excellent efficiencies of the duplex TaqMan-MGB qRT-PCR.

**Additional file 3 **: **Figure S3.** The amplification curves and the corresponding standard curve for detection of _g1_CZ73-M/_g1_AH320-M gene (A, B), and _g1_CZ73-PB2/_g1_AH320-PB2 (CD) gene showed excellent efficiencies of the duplex TaqMan-MGB qRT-PCR.

**Additional file 4 **: **Figure S4.** The specificity of duplex MGB TaqMan-probe-based real-time RT-qPCR. The M/PB2 genes from GD15, CZ73 and AH320 can’t be detected by SH14-Mprobe974 or SH14-PB2probe713, and M/PB2 genes from SH14 virus can’t be detected by GD15-Mprobe974, Mprobe975–17, GD15-PB2probe713 or PB2probe974RC, either.

## Data Availability

The datasets generated and analyses during the current study are available from the corresponding author on request.

## Abbreviations

293 T cells: Human embryonic kidney
MDCK cells: Madin-Darby canine kidney cells
DMEM: Dulbecco’s minimum essential medium
FBS: Fetal bovine serum
TCID50: The 50% tissue culture infection dose
MOIs: Multiplicity of infection
PBS: Phosphate-buffered saline
