# Supplementary figures and images for: G1-like M and PB2 genes are preferentially incorporated into H7N9 progeny virions during genetic reassortment
Source: BMC Vet Res. 2021 Feb 15;17:80. doi: 10.1186/s12917-021-02786-0 (PMC7885445; doi:10.1186/s12917-021-02786-0)

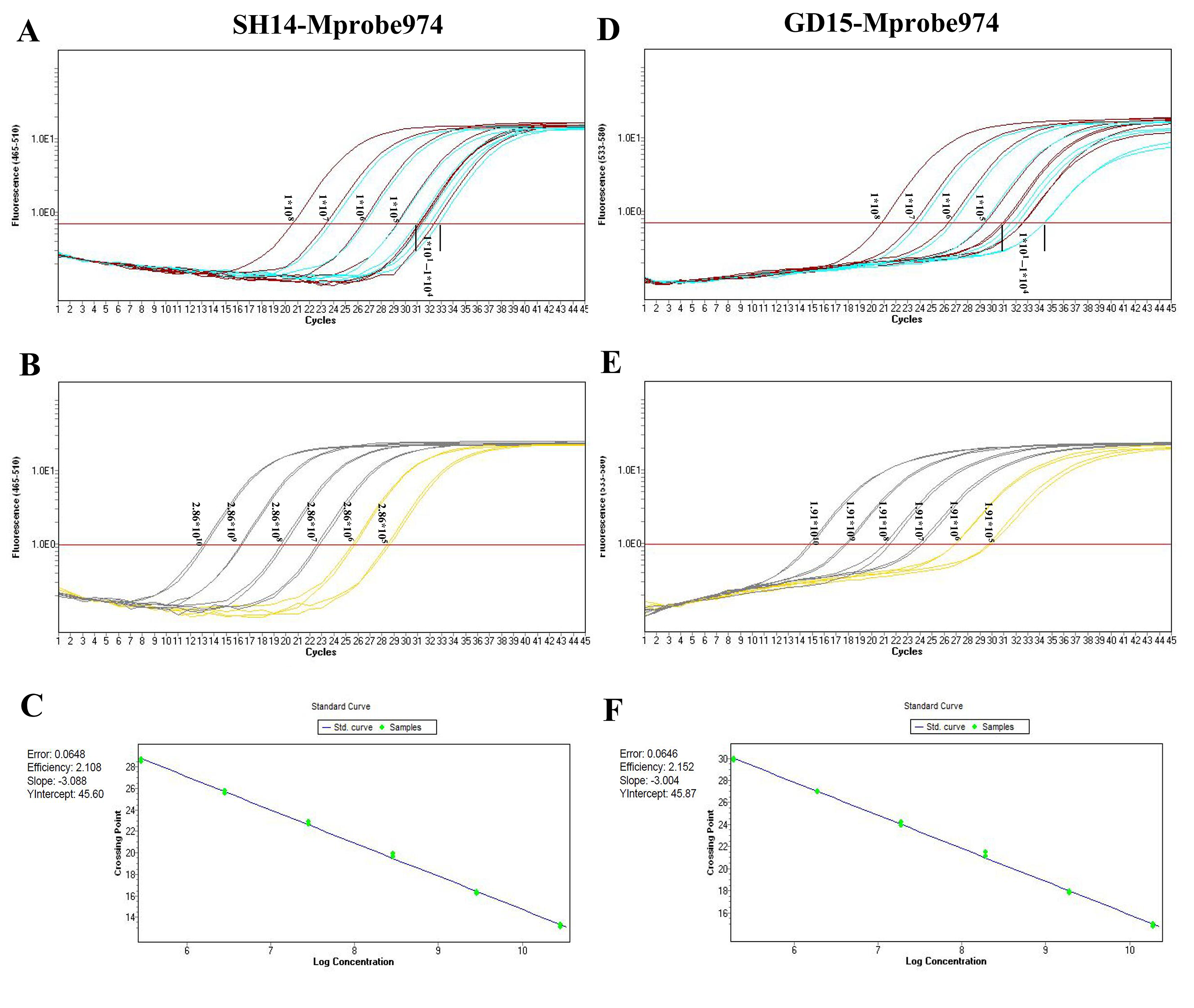

Supplement: Supplementary file 1 — Additional file 1 : Figure S1. Sensitivities and standard curves of the duplex TaqMan-MGB qRT-PCR targeting the M genes of SH14 and GD15 viruses. Sensitivities of SH14-Mprobe974 (A) and GD15-Mprobe974 (D) were detected by 10-fold serial dilutions of 108copies/ml f98SH14-M plasmid and g1GD15-M plasmid, respectively. The detection limit was approximately 10 copies of both gene when cp < 35. The amplification curves and the corresponding standard curve for detection of f98SH14-M (B, C) and g1GD15-M (E, F) showed excellent efficiencies of the duplex TaqMan-MGB qRT-PCR. [file 12917_2021_2786_MOESM1_ESM.tif]

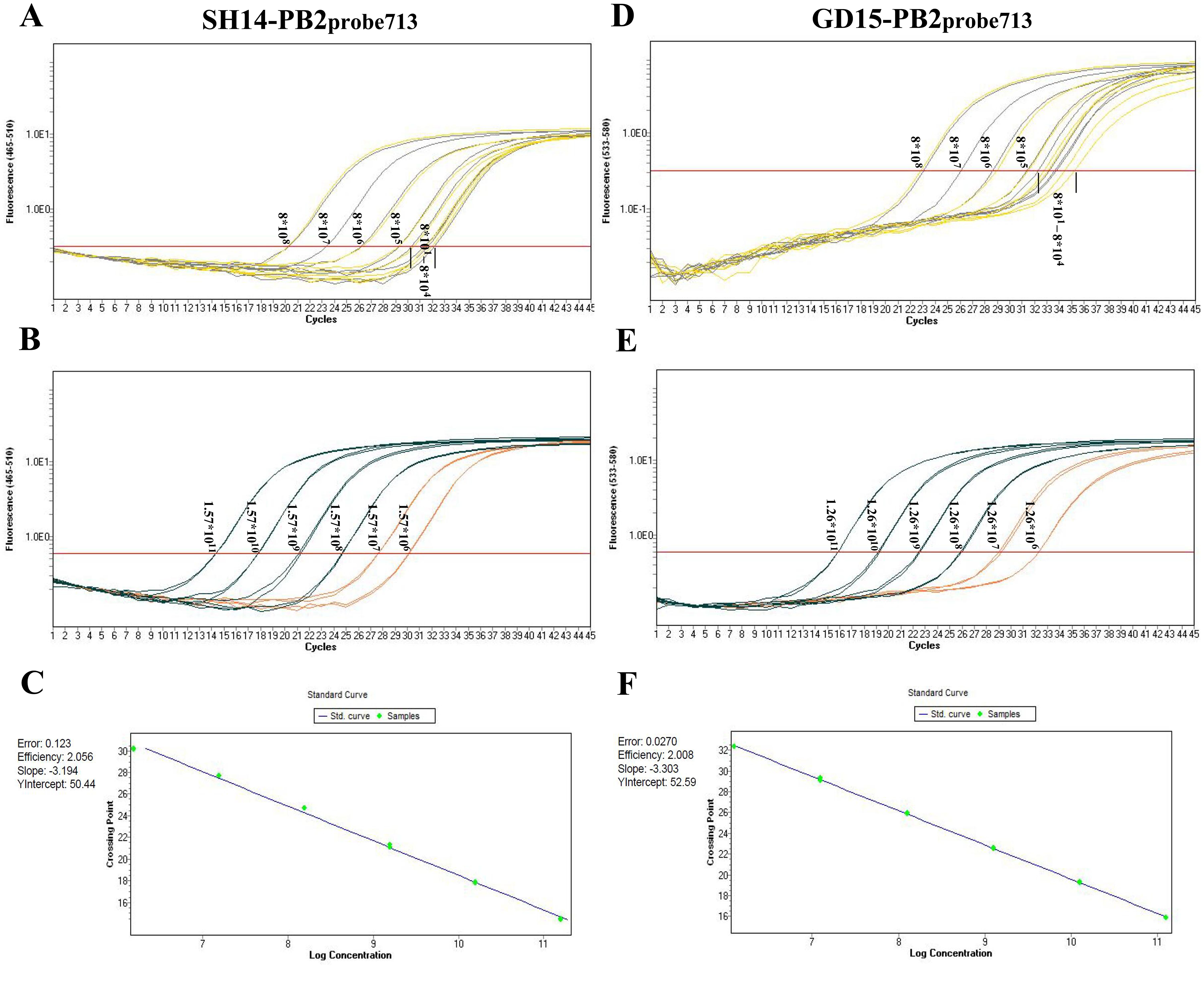

Supplement: Supplementary file 2 — Additional file 2 : Figure S2. Sensitivities and standard curves of the duplex TaqMan-MGB qRT-PCR targeting the PB2 genes of SH14 and GD15 viruses. Sensitivities of SH14-PB2probe713 (A) and GD15-PB2probe713 (D) were detected by 10-fold serial dilutions of 8*108copies/ml f98SH14-PB2 plasmid and g1GD15-PB2 plasmid, respectively. The detection limit was approximately 10 copies of both gene when cp < 35. The amplification curves and the corresponding standard curve for detection of f98SH14-PB2 (B, C) and g1GD15-PB2 (E, F) showed excellent efficiencies of the duplex TaqMan-MGB qRT-PCR. [file 12917_2021_2786_MOESM2_ESM.tif]

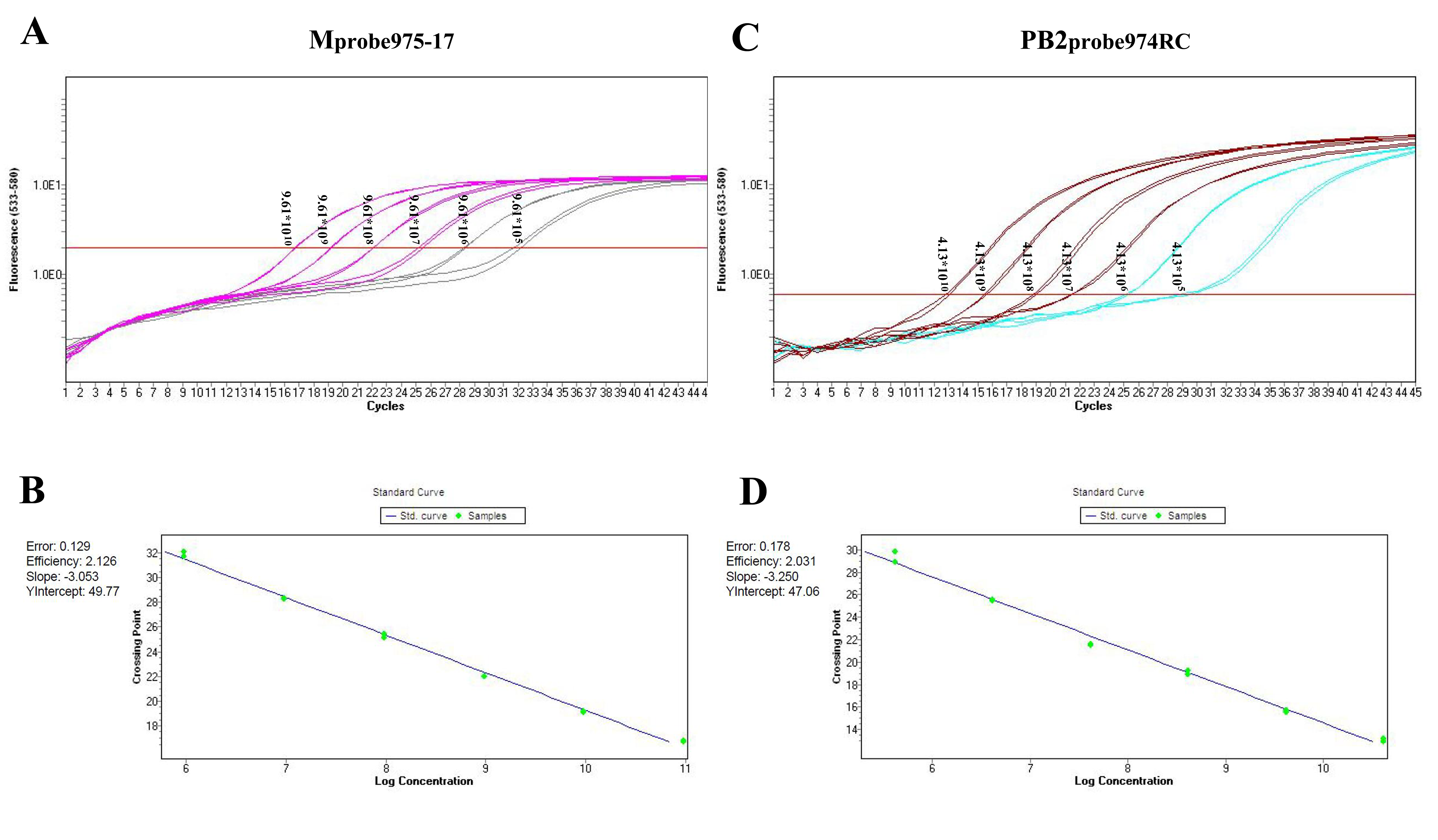

Supplement: Supplementary file 3 — Additional file 3 : Figure S3. The amplification curves and the corresponding standard curve for detection of g1CZ73-M/g1AH320-M gene (A, B), and g1CZ73-PB2/g1AH320-PB2 (CD) gene showed excellent efficiencies of the duplex TaqMan-MGB qRT-PCR. [file 12917_2021_2786_MOESM3_ESM.tif]

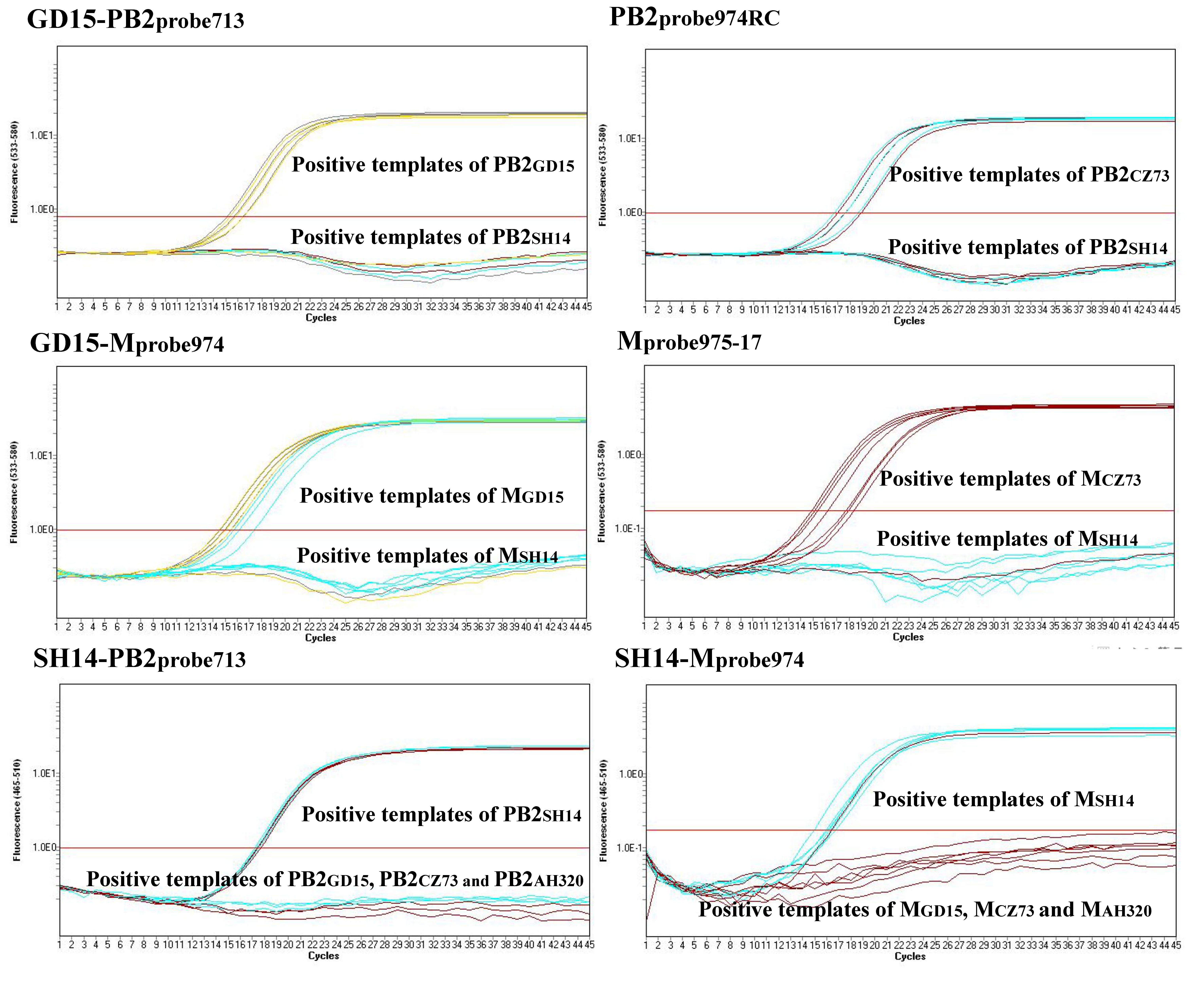

Supplement: Supplementary file 4 — Additional file 4 : Figure S4. The specificity of duplex MGB TaqMan-probe-based real-time RT-qPCR. The M/PB2 genes from GD15, CZ73 and AH320 can’t be detected by SH14-Mprobe974 or SH14-PB2probe713, and M/PB2 genes from SH14 virus can’t be detected by GD15-Mprobe974, Mprobe975–17, GD15-PB2probe713 or PB2probe974RC, either. [file 12917_2021_2786_MOESM4_ESM.tif]
